# Rapid detection of lung cancer based on serum Raman spectroscopy and a support vector machine: a case-control study

**DOI:** 10.1186/s12885-024-12578-y

**Published:** 2024-07-02

**Authors:** Linfang Yan, Huiting Su, Jiafei Liu, Xiaozheng Wen, Huaichao Luo, Yu Yin, Xiaoqiang Guo

**Affiliations:** 1 Guang’an People’s Hospital, Guang’an, Sichuan Province China; 2 Sichuan Cancer Hospital & Institute, Sichuan Cancer Center, Chengdu, China; 3 Sichuan Institute for Brain Science and Brain-Inspired Intelligence, MOE Key Lab for Neuroinformation, University of Electronic Science and Technology of China, Chengdu, China

**Keywords:** Raman spectroscopy, Lung cancer, Serum, SVM

## Abstract

**Background:**

Early screening and detection of lung cancer is essential for the diagnosis and prognosis of the disease. In this paper, we investigated the feasibility of serum Raman spectroscopy for rapid lung cancer screening.

**Methods:**

Raman spectra were collected from 45 patients with lung cancer, 45 with benign lung lesions, and 45 healthy volunteers. And then the support vector machine (SVM) algorithm was applied to build a diagnostic model for lung cancer. Furthermore, 15 independent individuals were sampled for external validation, including 5 lung cancer patients, 5 benign lung lesion patients, and 5 healthy controls.

**Results:**

The diagnostic sensitivity, specificity, and accuracy were 91.67%, 92.22%, 90.56% (lung cancer vs. healthy control), 92.22%,95.56%,93.33% (benign lung lesion vs. healthy) and 80.00%, 83.33%, 80.83% (lung cancer vs. benign lung lesion), repectively. In the independent validation cohort, our model showed that all the samples were classified correctly.

**Conclusion:**

Therefore, this study demonstrates that the serum Raman spectroscopy analysis technique combined with the SVM algorithm has great potential for the noninvasive detection of lung cancer.

## Background

Lung cancer has the highest incidence rate and mortality among all malignant tumors [1]. As the stage of lung cancer progresses, the five-year survival rate gradually decreases [2]. In stage IA groups, the rates exceed 90%, while in stage IV groups, the rates are less than 10% [2]. Therefore, the early diagnosis of lung cancer is of great significance and can considerably improve lung cancer patients’ therapeutic effects and prognosis. In the lung cancer screening guidelines, low-dose spiral computed tomography (LDCT) is recommended for people with high-risk factors worldwide [3]. However, in addition to radiation injury, the high false positive rate of LDCT will lead to unnecessary invasive examination and overdiagnosis [4, 5]. Pathological examination is the gold standard for lung cancer diagnosis. This technique commonly requires the help of surgical approaches such as fiber bronchoscopy, image-guided trans-thoracic needle aspiration, and thoracoscopy [6]. Additionally, these procedures are costly, prone to complications, and there is a possible need for enough samples [6]. Still, it is unsuitable for early lung cancer diagnosis due to the inescapable invasiveness and harsh application conditions. Routine serum biomarkers are unsatisfactory due to their lower sensitivity or specificity [7–9]. Liquid biopsy is not widely used in clinical practice due to the uncertainty threshold and high testing costs [10]. Thus, a more convenient and noninvasive diagnosis tool with high sensitivity and specificity is needed to reduce mortality rates and burdens on the medical system.

Raman spectroscopy is a well-established analytical technique based on the inelastic scattering generated by rotational and vibrational modes of molecular bonds [11]. Compared with routine diagnostic methods, Raman spectroscopic techniques have the advantages of being fast, accurate, and non-destructive [12]. Serum is mainly composed of water, carbohydrates, proteins, phospholipids and polysaccharides, showing a unique Raman fingerprint profile. Metabolism of malignant cells affect the composition and content change of serum [13, 14]. Raman-based methods can effectively detect minor changes that occur during cancer development [15]. Meanwhile, serum samples are easier to obtain and the Raman detection system has the advantages of no sample preparation and non-contact measurements. Effectiveness and safety of this detection method in the previous study about the COVID-19 has been confirmed [16]. Furthermore, a review about the potential of Raman spectroscopy to analyze liquid plasma/serum shows that liquid form has potential advantages over the infrared absorption analysis of dry droplet form and will prove to be highly beneficial to clinicians for rapid screening in the future [17]. More studies have found that Raman spectroscopy has diagnostic potential in a variety of tumors [18], while few original clinical trials utilized serum Raman spectroscopy to diagnose lung cancer. Therefore, investigations on the performance of serum Raman spectroscopy in diagnosing lung cancer are of great significance.

Due to the complexity and heterogeneity of Raman spectrum data, machine learning methods are necessary for deep data mining. SVM is a machine learning algorithm that classifies data based on supervised learning, particularly suitable for small sample problems and high latitude pattern recognition [19, 20]. SVM is an effective classifier because it can be used for both linearly separable and linearly inseparable data sets [21]. Additionally, the SVM algorithm is applied most frequently in classification and prediction methods with high accuracy for disease risk prediction [22]. Notably, the combination of SVM and Raman spectroscopy has previously been used to distinguish patients with hysteromyoma and cervical cancer from healthy controls and the results were satisfactory [23].

In this study, we detected the serum from lung cancer patients, benign lung lesion patients, and healthy controls using Raman spectroscopy to explore the screening value of Raman spectroscopy. Furthermore, a support vector machine (SVM) was used for model building and training.

## Methods

### Patients

A total of 90 patients with lung-occupying lesions were recruited in this project. Patients were selected and enrolled upon confirmation of clinical or pathological diagnosis. All enrolled participants at the time of pathological or clinical diagnosis of benign lung lesion or lung cancer reported no history of malignancy or prior treatment, such as chemotherapy or radiotherapy. The lung cancer group included patients with a pathological diagnosis of lung cancer. The stages were determined in accordance with the 8th edition of tumor node metastasis (TNM) classification [24] for lung cancer, with each lesion being individually staged and the final stage being based on the highest stage. In contrast, the benign lung lesion group included patients diagnosed with inflammatory or granulomatous changes in pathology. Thus, the lung cancer and benign lung lesion groups obtained 45 patients, respectively. The healthy control group also consisted of 45 healthy individuals recruited from the medical examination center of the participating institutes.

This study was approved by the Medical Ethics Committee of Guang’an People’s Hospital(approval number: 2,022,007), Sichuan Province, China, and conducted following the principles of the Declaration of Helsinki. All the enrolled individuals signed the written informed consent.

### Sample preparation

Fasting venous blood samples were collected from all participants before treatment. The serum was isolated from blood samples by centrifuging for 10 min at 3000 rpm. All the serum samples were strictly sealed in cryopreservation tubes and stored at -80 °C until being scanned. For the measurement, approximately 0.5 ml of the serum sample was prepared in cryopreservation tubes made of polypropylene with a specification of 2 ml. All blood samples were collected from March to December 2022 in this study.

### Raman measurements

The Raman system is designed by the Sichuan Institute for Brain Science and Brain-Inspired Intelligence, which consists of a volume-phase holographic spectrograph (F/2ctroEMvision), deep-cooled CCD camera (at -60 °C, Andor iVac DR-316B-LDC-DD), Raman probe, and laser. The sample end uses a microscope objective (50X, NA 0·5, WD 8·0, Sunnyoptical) as a focusing lens. An internal laser line filter (Semrock, LL01-785-12·5) was applied to obtain a clean laser profile. A single-mode diode laser with wavelength 785 nm and 100 mW power was used for Raman excitation. The laser power on the sample was detected to be around 70mW. Furthermore, the spectra were recorded in the 400–1800 cm-1 range. The detection process was repeated 3 times and acquired 5 spectra each time, and 15 Raman spectra were collected in total from each serum sample.

First, the ethanol spectrum was measured using an exposure time of 3s for the wavenumber calibration. Second, the cryopreservation tubes with a 5% normal saline spectrum were acquired using an exposure time of 3 s with five successive scans for every beginning and completion of the experiment. The average spectrum of the cryopreservation tube spectra was used for background subtraction. Next, the Raman spectrum of the serum samples sealed within the cryopreservation tube was measured using the same integration parameters as the cryopreservation tube measurements. Three experimenters took the Raman scan for each sample tube and repeated it five times. Following cosmic ray removal from the spectral data, we had 15 scans per serum sample. Furthermore, the cryopreservation tube was placed in the specific card slot of the Raman spectrometer, ensuring that the laser passed through the tube wall at a certain angle.

### Date processing and SVM classification

Raman spectral data preprocessing steps include smoothing by automatic-weighted least squares, baseline correction based on polynomial fitting, and normalization by total area. A total of 1800 spectra from 120 individuals were preprocessed for model building. The ANOVA statistical test method was used to select relevant features. Additionally, the points that passed the ANOVA statistical test between the two groups were used as input features of the SVM. Our SVM algorithm used a non-linear radial basis function (RBF).

In this study, we used a two-level cross-validation approach. The model building data set were divided into two groups, containing 70% and 30% of the data for training and testing groups, respectively. The training and cross-validation data sets were separated by randomly selecting 70% of the total data. The remaining 30% of the data was used as unseen data to assess the predictive power of the classification models. The process mentioned above was repeated 50 times.

We externally validated the predictive model after it was built. In total, 15 serum samples were selected for verification, including 5 lung cancer patients, 5 patients with benign lung lesions, and 5 healthy controls. These samples are used as independent external datasets, and their spectra were preprocessed the same way as those used to build the model. Using an SVM model, each unlabeled spectrum was assigned to the class with the highest probability. The true classification of the samples was not revealed until after the model had made its predictions, allowing for an unbiased evaluation of the model’s performance. Finally, the receiver operating curve (ROC) was used to check the diagnostic performance of the model. MATLAB was used for the preprocessing of Raman spectrum data and the calculation of SVM and ROC.

## Results

### Clinical characteristics

Data regarding the age, sex, pathological results, and clinical stages of the participants are shown in Table 1. There is no statistically significant difference between these three groups regarding gender and age. The lung cancer group consisted of 20 cases of adenocarcinoma, 14 cases of squamous cell carcinoma, 8 cases of small cell carcinoma, 1 case of neuroendocrine tumor, 1 case of adenosquamous cell carcinoma, and 1 case of undifferentiated carcinoma. Moreso, the benign lung lesion group included 16 cases of pneumonia, 14 cases of chronic obstructive pulmonary diseases, 11 cases of tuberculosis, 2 cases of benign lung tumor, 1 case of interstitial lung disease, and 1 case of fungal pneumonia.

**Table 1:** Clinical characteristics

	Lung cancer	Benign lung lesion		Healthy control
	*n* = 45	*n* = 45		*n* = 45
Age, y				
Min-max(median)	51–85(67)		55–84(67)	56–76(65)
Sex				
Male	35		29	27
Female	10		16	18
Pathological findings				
Adenocarcinoma	20			
Squamous cell carcinoma	14			
Small cell carcinoma	8			
Neuroendocrine tumor	1			
Adenosquamous carcinoma	1			
Undifferentiated carcinoma	1			
TNM Stage				
I	12			
II	9			
III	8			
IV	16			

### Raman Spectra and statistical analysis

**Fig. 1:**
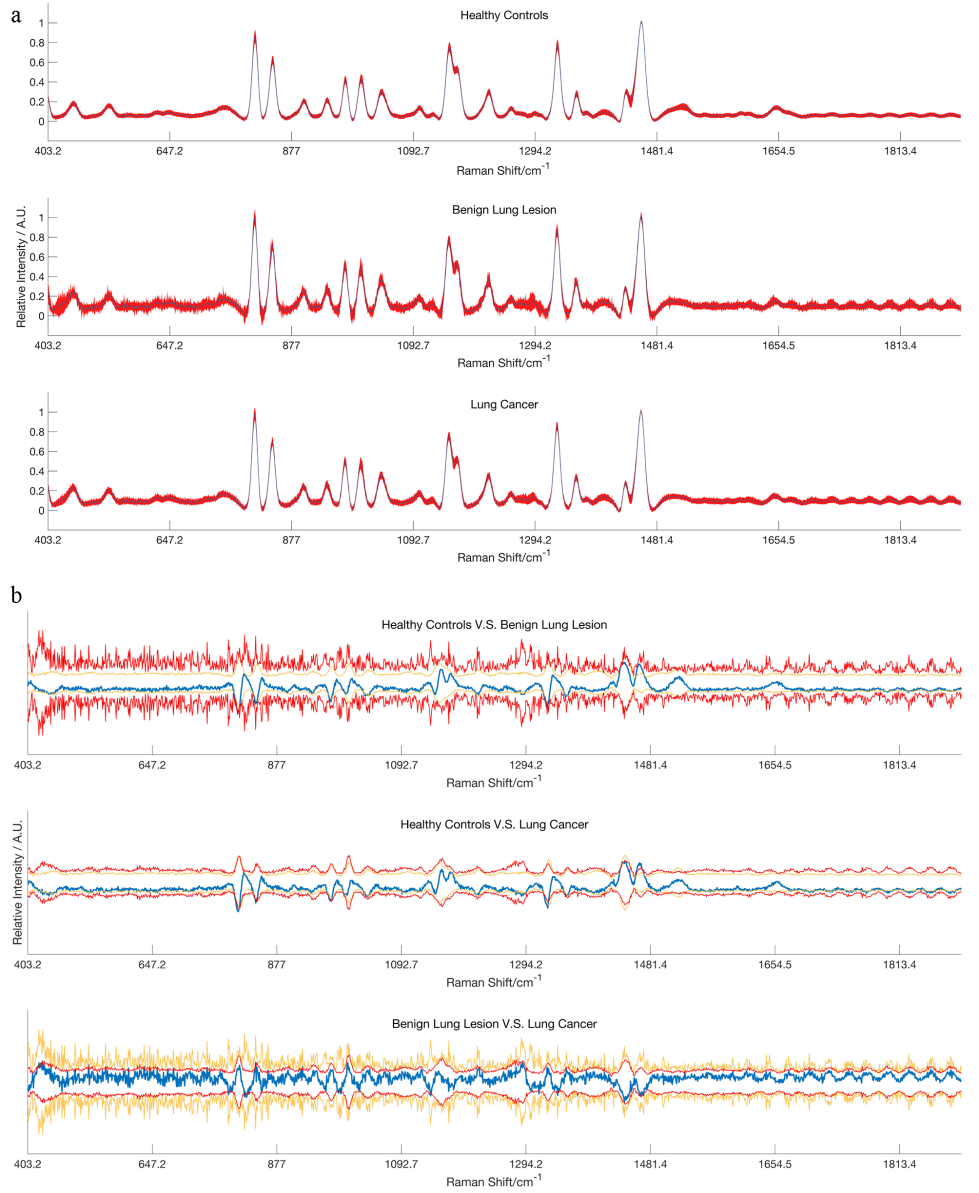
The total average serum Raman of the three groups and the difference between the groups. **(a)** The total average of the three types of Raman, the color band represents the standard deviation. **(b)** The Raman difference signal between the groups (blue) and the Raman signal of the groups between ± 2 standard deviations (red and yellow)

The mean preprocessed spectra of the three groups are shown in Fig. 1a. The differences between healthy controls vs. benign lung lesions, healthy controls vs. lung cancer, and benign lung lesions vs. lung cancer are depicted in Fig. 1b. The difference in the mean spectrum is shown within ± 2 standard deviations, suggesting that the mean difference between the groups is statistically insignificant. It is necessary to exploit the difference that may exist through deep learning algorithms.

**Fig. 2:**
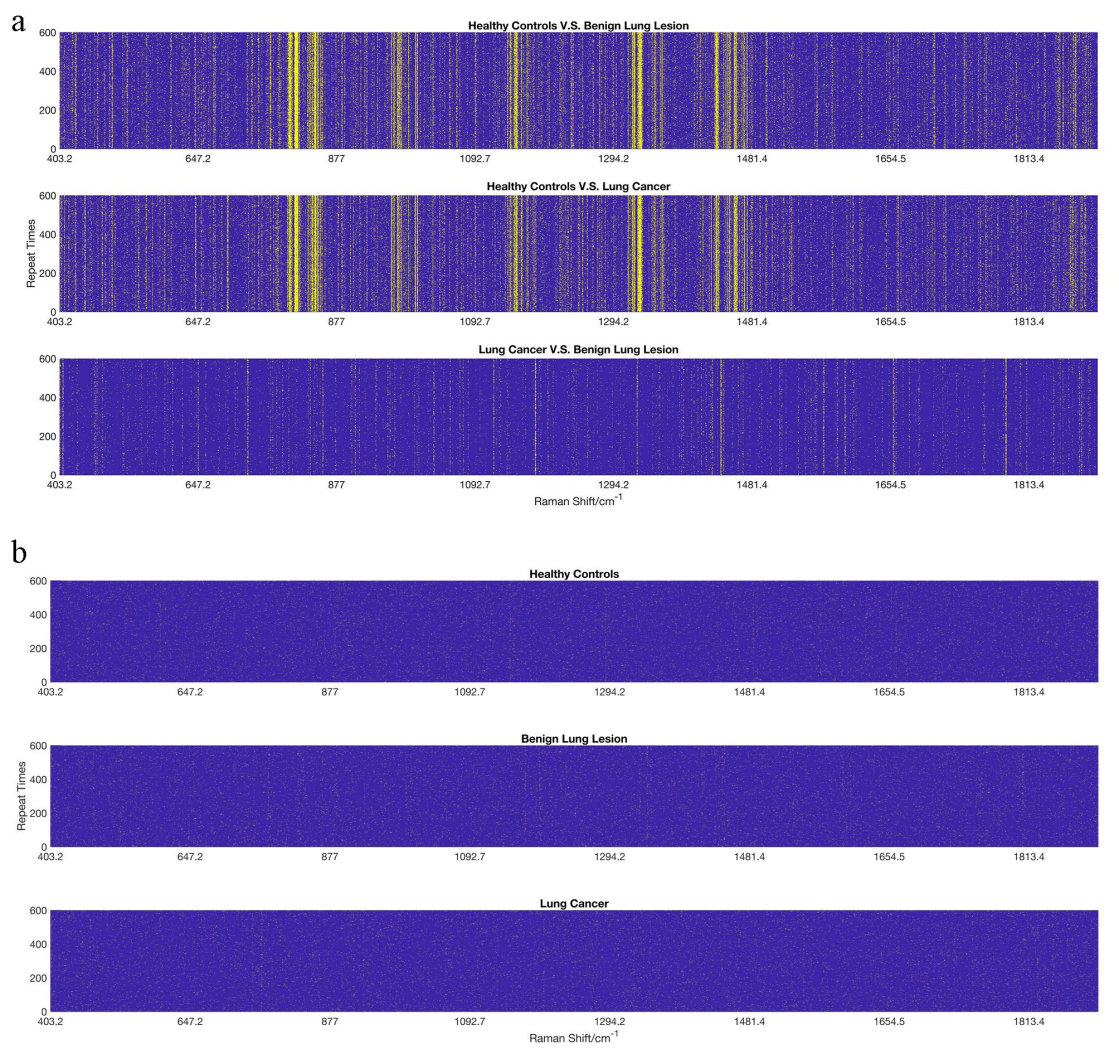
The result of the ANOVA test. The spectra range without a significant difference in the ANOVA test (*p* < 0.05) was indicated in blue, while others were indicated in yellow. **(a)** The Raman shift spectrum of the difference in the *p*-value for the inter-group. **(b)** The Raman shift spectrum of the difference in the *p*-value for the intra-group

The ANOVA test selected relevant features between the three experimental groups. The sample selection process was random and needed to be repeated 100 times. Only the statistical significance of the ANOVA test over 70 times out of 100 points was selected as the feature. High inter-group consistency was shown after the ANOVA analysis, while the differences in the intra-group were random. The difference between the three compared groups was in the spectra range of 400–1800 cm − 1 and is observed in Fig. 2a. The differences between lung cancer vs. benign lung lesions shown are significantly less than those two compared groups. However, the ANOVA test result showed no apparent consistency for the inter-group (Fig. 2b).

**Fig. 3:**
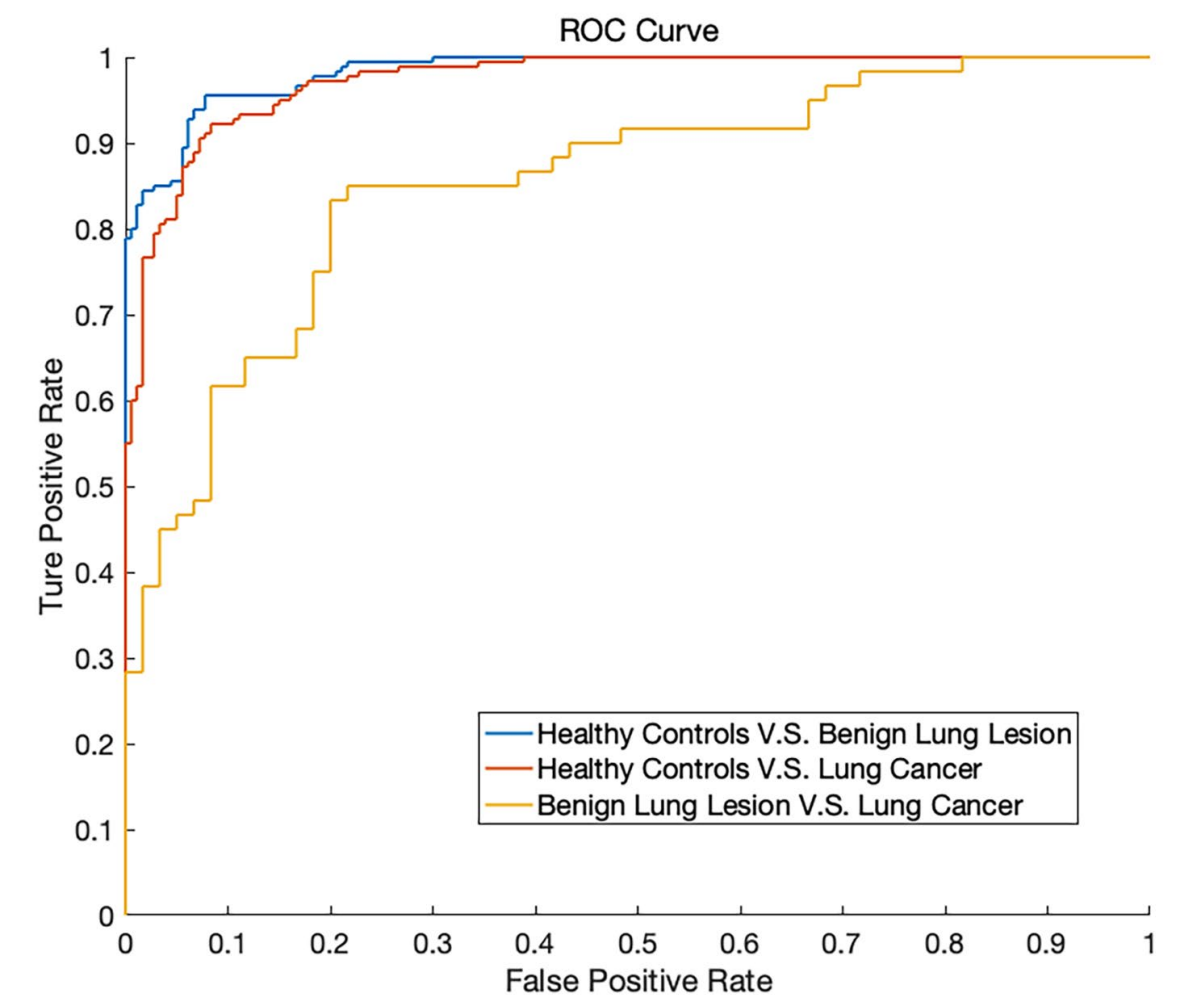
The ROC curve of the SVM diagnostic algorithm for the healthy controls group vs. benign lung lesion group, healthy controls group vs. lung cancer group, and benign lung lesion group vs. lung cancer group

Serum Raman spectroscopy combined with support vector machine algorithm shows great diagnostic ability in lung cancer screening. The performance of the classifiers are evaluated by the ROC curve and shown in Fig. 3. All ROC analyses are based on nonparametric techniques and are conducted for the SVM analyses. For each of the three classification tasks, the area under the curve (AUC) value and the results of AUC, specificity, accuracy, and sensitivity of the SVM classification are calculated and shown in Table 2.

**Table 2:** Performance parameters of the SVM

Class	Value ± SD			
	AUC	Sensitivity	Specificity	Accuracy
healthy controls vs. benign lung lesions	0.984 ± 0.003	0.922 ± 0.004	0.956 ± 0.002	0.933 ± 0.006
healthy controls vs. lung cancer	0.974 ± 0.003	0.917 ± 0.007	0.922 ± 0.006	0.906 ± 0.005
lung cancer vs. benign lung lesions	0.853 ± 0.006	0.800 ± 0.007	0.833 ± 0.005	0.808 ± 0.011

The overall serum-level classification of each of the 15 serum samples is shown in Table 3. A serum sample was assigned to the class that received the majority of spectra assigned to it. For the independent test, our model showed that all the samples were classified correctly.

**Table 3:** Results of 15 samples for the independent verification

Individual spectra predictions						
Sample #	Number of prediction			External validation results		
	Healthy control	Benign lung lesion	Lung Cancer	Sample #	Predicted class	True class
1	990	307	203	1	Healthy Control	Healthy Control
2	985	375	140	2	Healthy Control	Healthy Control
3	989	313	198	3	Healthy Control	Healthy Control
4	994	200	306	4	Healthy Control	Healthy Control
5	997	300	203	5	Healthy Control	Healthy Control
6	237	911	352	6	Benign lung lesion	Benign lung lesion
7	319	949	232	7	Benign lung lesion	Benign lung lesion
8	212	982	306	8	Benign lung lesion	Benign lung lesion
9	345	986	169	9	Benign lung lesion	Benign lung lesion
10	299	982	219	10	Benign lung lesion	Benign lung lesion
11	98	695	707	11	Lung Cancer	Lung Cancer
12	235	294	971	12	Lung Cancer	Lung Cancer
13	208	316	976	13	Lung Cancer	Lung Cancer
14	276	266	958	14	Lung Cancer	Lung Cancer
15	203	488	809	15	Lung Cancer	Lung Cancer

## Discussion

Raman spectroscopy measurement is an increasingly popular method of diagnosing cancer [18]. Recently, many studies have shown that Raman spectroscopy is a high-accuracy method for diagnosing lung cancer [25–30]. However, most studies mainly carried out Raman detection on tissues to screen for lung cancer [27–30]. Raman detection using tissue is not as convenient as blood detection in general physical examination. Notably, serum detection could be a more favorable and noninvasive method than tissue. Once lung cancer screening can be carried out through blood testing, early lung cancer screening can be realized in a general physical examination which is incomparable with tissue testing [17].

Our study observed significant differences between the average Raman spectrum of lung cancer patients and healthy controls. Meanwhile, the classification model of lung cancer patients and healthy controls show excellent discrimination ability with AUC values of 0.973, and the sensitivity and specificity were 0.917 and 0.922, respectively. Similar conclusions of serum samples detected by Raman spectroscopy were also produced in Shin et al. [31]. and Moisoiu et al. [32]. studies in which diagnostic sensitivity and specificity in lung cancer were 0.84 (95% CI 0.69–0.93), 0.85 (95% CI 0.62–0.97) and 0.85 (95% CI 0.68–0.95), 0.87 (95% CI 0.73–0.96), respectively. Moreso, Lei et al. [33]. used surface enhanced Raman spectroscopy (SERS) combined with principal component analysis (PCA) and partial least-squares discriminant analysis (PLS-DA) to diagnose and distinguish lung cancer and normal serum. Importantly, the model’s sensitivity improved to 100%, while the specificity decreased to 83.33% [29]. Compared with these similar studies, our classification model seems more excellent. From the studies of Ke et al. [34]. and Chen et al. [35]. , the results of tissue samples detected by Raman spectroscopy were more reliable than serum samples. Tissue samples are certainly better, but they cannot be used for early screening of lung cancer because they are difficult to obtain. Therefore, if the pathology sample was unavailable, serum detection could be more favorable and noninvasive.

Different from previous studies, our study included a benign lung lesion group, which is also one of our innovative points. The majority of patients in the benign lung lesion group were diagnosed with infectious inflammation. There is a certain similarity between serum metabolites in cancers and inflammatory diseases [13, 14, 36]. Furthermore, most patients in our lung cancer groups are usually not challenged with a single disease, and they are often concurrent with chronic lung inflammatory disease. Therefore, the dual factors increase the classification difficulty of our model of lung cancer patients and benign lung lesion individuals. This result may be why our model’s diagnosis accuracy, sensitivity, and specificity were only 0.808, 0.800, and 0.833, respectively. From the lung cancer and benign lung lesion classification model, the area under the curve (AUC) value was also obiviously lower than that of the other two classification models, only 0.853. Nevertheless, our results are meaningful and provide a reference for differentiating benign and malignant lung diseases. Takamori et al. analyzed salivary metabolites and built a multiple logistic regression (MLR) models for discriminating patients with lung cancer from benign lung lesions (AUC = 0.729, 95%CI = 0.598–0.861, *p* = 0.003) [37]. Compared with this consequence, our research shows a more robust diagnostic ability. Besides the use of cancer diagnosis, Raman spectroscopy has also been applied in many studies regarding inflammatory diseases such as dengue fever [38], malaria infection [39], virus infection [40], cryptococcal infection [41], ulcerative colitis [42], and cervicitis [43]. Our results are consistent with these studies and shows more excellent diagnostic ability. Therefore, our model has a good clinical practicability.

## Conclusions

This study is based on a label-free serum detection of the Raman spectrum and combined with machine learning methods to realize the rapid diagnosis of lung cancer. We used the SVM to establish the two-way (lung cancer vs. healthy control, benign lung lesion vs. healthy, and lung cancer vs. benign lung lesion) classification model. Notably, all three models demonstrated an outstanding differentiation ability. Therefore, these findings indicate that the serum Raman spectroscopy combined with a support vector machine model can be used as a standard prescreening tool for lung cancer.

This study also has limitation. The sample size was small, and well-powered large-scale multicenter studies are needed to verify this conclusion in the future. Besides, prospective early screening clinical trial design is also needed.

## Data Availability

The datasets used and analyzed during the current study are available from the corresponding author upon reasonable request.

## Abbreviations

SVM: Support vector machine
LDCT: Low-dose spiral computed tomography
TNM: Tumor node metastasis
ROC: Receiver operating curve
AUC: area under the curve
RBF: Non-linear radial basis function
SERS: Surface-enhanced Raman spectroscopy
PCA: Principal component analysis
PLS-DA: Partial least-squares discriminant analysis
